# Supplementary material for: Determinants of life expectancy in most polluted countries: Exploring the effect of environmental degradation
Source: PLoS One. 2022 Jan 21;17(1):e0262802. doi: 10.1371/journal.pone.0262802 (PMC8782287; doi:10.1371/journal.pone.0262802)
Supplement: S1 Appendix — (DOCX) [file pone.0262802.s001.docx]

**S1 Appendix**

**Table 6: The results of PCSE regression using time trend variable**

|  | PCSE |
| --- | --- |
| _Constant | -276.28 (18.58)^***^ |
| lnGDP | 1.73 (0.164)^***^ |
| lnCO_2_ | -0.69 (.010)^***^ |
| lnHEX | 0.64 (0.13)^***^ |
| lnWAT | 11.45 (0.39)^***^ |
| lnSAN | 1.85 (0.21)^***^ |
| Year | 0.13 (0.01)^***^ |
| R-squared | 0.9999 |
| Wald chi^2^ | 9081.9 |
| Probability | 0.0000 |
| N | 558 |

Note: ^***^ indicates significance at 1% level. Stanradr errors are in the peranthesis.

**Table 7: Robustness check: The results of FGLS regression using time trend variable**

|  | FGLS |
| --- | --- |
| Constant | -299.59 (79.58)^***^ |
| lnGDP | 1.19 (0.39)^***^ |
| lnCO_2_ | -0.55 (0.24)^***^ |
| lnHEX | 0.81 (0.34)^***^ |
| lnWAT | 11.80 (0.90)^***^ |
| lnSAN | 1.49 (0.53)^***^ |
| Year | 0.15 (0.04)*** |
| Wald chi^2^ | 1378.6 |
| Probability | 0.0000 |
| N | 558 |

Note: ^***^ indicates significance at 1% level. Stanradr errors are in the peranthesis.
