# Supplementary material for: Determinants of life expectancy in most polluted countries: Exploring the effect of environmental degradation
Source: PLoS One. 2022 Jan 21;17(1):e0262802. doi: 10.1371/journal.pone.0262802 (PMC8782287; doi:10.1371/journal.pone.0262802)
Supplement: S2 Appendix — (DOCX) [file pone.0262802.s002.docx]

**S2 Appendix**

| **Countries by order of pollution level** | **Pollution level (average PM2.5 (mg/m3))*** | **Life expectancy (Year)**** |
| --- | --- | --- |
| Bangladesh | 83.3 | 72.05 |
| Pakistan | 65.81 | 66.94 |
| Mongolia | 62 | 69.50 |
| Afghanistan | 58.8 | 64.13 |
| India | 58.08 | 69.16 |
| Indonesia | 51.71 | 71.28 |
| Bahrain | 46.8 | 77.03 |
| Nepal | 44.46 | 70.17 |
| Uzbekistan | 41.2 | 71.39 |
| China | 39.12 | 76.47 |
| UAE | 38.94 | 77.65 |
| Kuwait | 38.3 | 75.31 |
| Vietnam | 34.06 | 75.24 |
| Uganda | 29.1 | 62.52 |
| Bulgaria | 25.49 | 74.81 |
| Sri Lanka | 25.2 | 76.65 |
| Korea Republic | 24.78 | 82.63 |
| Iran | 24.27 | 76.27 |
| Thailand | 24.25 | 76.68 |
| Kazakhstan | 23.6 | 72.95 |
| Peru | 23.38 | 76.29 |
| Servia | 23.3 | 76.09 |
| Chile | 22.63 | 79.91 |
| Nigeria | 21.4 | 53.95 |
| Cambodia | 21.1 | 69.29 |
| Turkey | 20.62 | 77.16 |
| Ethiopia | 20.1 | 65.87 |
| Mexico | 20.02 | 74.95 |
| Croatia | 19.09 | 77.83 |
| Poland | 18.67 | 77.85 |
| Czech Republic | 14.45 | 79.47 |

* Data are based on World Population Review 2021. For our empirical analysis, data were based on World Population Review 2019, where average PM2.5 of all countries were more than 20 (our benchmark). During the last two years, pollution level reduced to below 20 for Croatia, Poland and Czech Republic.

** Data based on year 2017.
