# Supplementary material for: Determinants of life expectancy in most polluted countries: Exploring the effect of environmental degradation
Source: PLoS One. 2022 Jan 21;17(1):e0262802. doi: 10.1371/journal.pone.0262802 (PMC8782287; doi:10.1371/journal.pone.0262802)
Supplement: S3 Appendix — (DOCX) [file pone.0262802.s003.docx]

**S3 Appendix**

Pesaran [53] advocates the following Augmented Dickey-Fuller regression of the individual series:

$\Delta y_{it} =\alpha_{i}+ p_{i}\bar{y}_{t-1}+\sum_{j=0}^{k} \gamma_{ij}\Delta\bar{y}_{it-1}+ \sum_{j=0}^{k} \delta_{ij}y_{it-1}+\varepsilon_{it}$ (5)

where $\bar{y}_{t-1}$ is the cross-sectional averages of lagged levels and $\Delta\bar{y}_{it-1}$ is first differences individual series. Once running the CADF (covariate-augmented Dickey Fuller) statistics, the CIPS (cross sectionally augmented IPS) statistic can be obtained as follows:

$CIPS=\left( \frac{1}{N} \right)\sum_{i=1}^{N} t_{i}\left( N, T \right)$ (6)

Im, Pesaran and Shin (2003) suggest the t-bar test using the below equation.

$t-bar= \sqrt{N\left( t_{\propto}-k_{t} \right)}/\sqrt{v_{t}}$ (7)

where N is the panel size, t_α_ is the average of the individual ADFt- statistics for the cross-sectional unit. k_t_ and v_t_ are the estimates of the mean and variance of each t_αi_ statistics, respectively, which are generated by simulations; they tabulate exact critical values for different combinations of N in Im, Pesaran and Shin [56] test.

Fisher (57) augmented Dickey–Fuller (ADF) test is as follows:

$y_{t} ={\beta^{,}D}_{t}+ \phi y_{t-1}+\sum_{j=0}^{p} \psi_{ij}\Delta y_{t-j}+\varepsilon_{it}$ (8)

Where D_t_ is a vector deterministic term. ∆y_t-j_ are the p lagged difference terms, which are used to approximate the ARMA structure of the errors.

The Harris-Tsavalis test statistic based on the OLS estimator, p, in the regression model is:
$y_{it} ={py}_{i,t}+ z_{i,t}^{,}\gamma_{i} +\varepsilon_{it}$ (9)

where the term $z_{i,t}^{,}\gamma_{i}$ permits for panel means and trends.

Pesaran [58] suggests that if the cross-sectional size is greater than the time dimension, the following test statistic can be used instead.

$CD= \sqrt{\frac{2T}{N(N-1)}} \sum_{i=1}^{N-1} \sum_{j=i+1}^{N} {\hat{p_{ij}}}^{2}$ (10)

Where $\hat{p_{ij}}$ denotes a correlation between the errors. Here the null hypothesis is H_0_: no cross-sectional dependence against the alternative hypothesis H_1_: cross-sectional dependence.
