# Supplementary material for: Determinants of life expectancy in most polluted countries: Exploring the effect of environmental degradation
Source: PLoS One. 2022 Jan 21;17(1):e0262802. doi: 10.1371/journal.pone.0262802 (PMC8782287; doi:10.1371/journal.pone.0262802)
Supplement: Attachment — Submitted filename: Response to Reviewer & Editor- Plos One.docx [file pone.0262802.s004.docx]

03 December 2021

María del Carmen Valls Martínez, Ph.D.
Academic Editor
PLOS ONE

Re: Revised version of the paper entitled *Determinants of Life Expectancy in Most Polluted Countries: Exploring the Effect of Environmental Degradation* **----***Response to the Editor and Reviewers.*

**PONE-D-21-32920**

PLOS ONE

Dear Editor,

We are grateful to you for providing us the opportunity to revise the paper based on the recommendations of the reviewers. We are especially thankful to our respected reviewers for their thoughtful and constructive feedback on the paper. We very much appreciate for the time they spent on the paper. We have tried our best to accommodate/address all the suggestions they made in the paper. Because of their help, and our subsequent actions on their feedback, we believe the quality of the paper is now much improved. Their Comments and our Actions/Responses are noted below in detail for your kind consideration.

| **Reviewer 1: Comments** | **Our Action and Responses** |
| --- | --- |
| Is the manuscript technically sound, and do the data support the conclusions?  The manuscript must describe a technically sound piece of scientific research with data that supports the conclusions. Experiments must have been conducted rigorously, with appropriate controls, replication, and sample sizes. The conclusions must be drawn appropriately based on the data presented.  Reviewer #1: Partly | Agree. Many thanks for the comment.  Revised version is much improved. We believe it satisfies this criterion. |
| Has the statistical analysis been performed appropriately and rigorously?  Reviewer #1: Yes | Many thanks for the comment. |
| Have the authors made all data underlying the findings in their manuscript fully available? Reviewer #1: Yes | Many thanks for the comment. |
| Is the manuscript presented in an intelligible fashion and written in standard English? | Agree. Many thanks for the comment. |
| Reviewer #1: First of all, I would like to thank the editorial board of Plos One for their confidence in my tasks as a reviewer. Regarding the review of the paper "Determinants of Life Expectancy in Most Polluted Countries: Exploring the Effect of Environmental Degradation", Manuscript Number: PONE-D-21-32920, the following is the outcome of my review:  Major Revision  In my opinion, this is not a bad paper at all. However, there are a number of weaknesses that do not recommend its acceptance in its current state. In this regard, I will indicate below a series of changes and proposals for improvement that I recommend that should be carried out with a view to a plausible acceptance of the article. | Many thanks for the comment.  Agree. We made required changes. Updated the manuscript as required. |
| Background section. I consider that this work lacks an introductory section that begins the work, stating its objectives, achievements and scope, ending with a brief paragraph indicating the topics to be developed in each of the subsequent subsections and the hypothesis to be verified. | Many thanks for the comment. Updated the background section.  Section 1.Page 4 to 7.  Renamed the section as ‘Introduction’ based on the suggestion of reviewer 2. |
| It is also necessary for the background section to include a more elaborate state of the question than the one presented in this paper in order to contextualize and conceptualize the term "Life Expectancy" in a balanced way. On the other hand, this work omits some previous articles, some of them already classics such as the ones I indicate below, which I strongly suggest to include as a bibliographic basis for this paper:   o Auster, R., Levesoardln, I. and Sarachek, S. (1969), 'The production of health: An exploratory study', J. Hum. Resour 4, 411-436.  o Crémieux, P.-Y., Ouellette, P. and Pilon, C. (1999), 'Health care spending as determinants of health outcomes', Health Econ 8, 627-639.  o Crémieux, P., Mieilleur, M., Ouellette, P., Petit, P., Zelder, P. and Potvin, K. (2005), 'Public and private pharmaceutical spending as determinants of health outcomes in Canada', Health Econ 14, 107-116.  o Halicioglu, F. (2011), 'Modeling life expectancy in Turkey', Econ. Model 28, 2075-2082.  o Hitiris, T. and Posnett, J. (1992), 'The determinants and effects of health expenditure in developed countries', J. Health Econ 11, 173-181.  o Martín Cervantes, P. A., Rueda López, N. and Cruz Rambaud, S. (2019), 'A Causal Analysis of Life Expectancy at Birth. Evidence from Spain', International Journal of Environmental Research and Public Health 16(13), 2367.  o Martín Cervantes, P. A., Rueda López, N. and Cruz Rambaud, S. (2020), 'The Relative Importance of Globalization and Public Expenditure on Life Expectancy in Europe: An Approach Based on MARS Methodology', International Journal of Environmental Research and Public Health 17(22), e8614.  o Thornton, J. (2002), 'Estimating a health production function for the US: Some new evidence', Appl. Econ 34, 59-62.  o Wolfe, B. and Gabay, M. (1987), 'Health status and medical expenditures: More evidence of a link', Soc. Sci. Med 25, 883-888. | Agree. Many thanks for the comment.  Updated the manuscript.  Section 1.1, Page 7 to 11.  Cited all the recommended papers in the manuscript.  Section 1.1 |
| Data  Please create a table with the 31 countries selected in your study and indicate the following, ordered from highest to lowest: Country-Pollution degree-Life expectancy years.  On the other hand, what is the reason for choosing "PM2.5 (mg/m3), an air pollutant, is greater than 20? Was this choice made on the basis of any previous work? Does it find any support in the literature or is it a criterion freely used by the authors? Please specify.  Regarding the table with descriptive statistics, perfect, but make a minimum comment on these statistics. | Many thanks for the comment. The list of countries as per suggestion is shown as Appendix B.  The authors set two criteria for selecting 31 countries: (i) minimum pollution level, "PM2.5 (mg/m3), an air pollutant, is greater than 20, and (ii) data availability considered in the study. Some countries with pollution level higher than 20 were not included due to many missing data. |
| Panel data unit tests  To be frank, I do not see any logic or usefulness in developing the formulas for the various tests used. In a textbook probably yes, but not in a scientific article. Therefore, I recommend eliminating such development or moving it to an appendix section. | Many thanks for the comment.  These are now removed, and shown as Appendix C. |
| In the same way, I do consider it necessary to additionally perform the KPSS test (Kwiatkowski-Phillips-Schmidt-Shin (KPSS)) since it would supplement your results from an alternative point of view, taking into account that contrary to most unit root tests, the presence of a unit root is per se not the null hypothesis but the alternative hypothesis. | Thanks for the comment. KPSS test (Kwiatkowski-Phillips-Schmidt-Shin (KPSS, 1992) is used for stationarity of a time series analysis (<https://en.wikipedia.org/wiki/KPSS_test>  ). Since our study is based on panel data, we believe this test is not applicable for this research. |
| Please include the Granger causality test among the citations used and, of course, include the limitations found in the conclusions. | Many thanks for the comment. Granger causality test is performed, and results are noted and discussed (see pp. 22-23) |
| The results obtained are quite good and congruent. It can be seen that the 31 countries selected are low-middle income countries. Please note this fact and, of course, include it in the discussion section. On the other hand, the discussion section is excessively sparse; please explain it with much more detail. The results obtained in the light of the literature should be presented in much greater depth, using also the suggested papers and the new section devoted to the literature review that you have to prepare. | Thanks for the comments. The results and discussion sections are re-written as per the suggestions. |
| Once you make each and every one of the suggested changes, I would be delighted if this work is finally accepted.  With my best wishes in your personal and academic life, | Thanks a lot for the comment. All the required changes are done as per the suggestion. |
|  |  |
| **Reviewer 2: Comments** | **Our Action and Responses** |
| Is the manuscript technically sound, and do the data support the conclusions?  Reviewer #2: Partly | Many thanks for the comment. |
| Has the statistical analysis been performed appropriately and rigorously?  Reviewer #2: Yes | Many thanks for the comment. |
| Have the authors made all data underlying the findings in their manuscript fully available?  Reviewer #2: No | Many thanks for the comment. Data will be available on request. Moreover, these data are freely available; data sources are noted in the manuscript. Anyone can freely view those data from the given sources. |
|  |  |
| I found the article very interesting and very well done from a methodological point of view. | Many thanks for the comment. |
| The first section should be called Introduction and should be the introduction, not the literature review. This section should include all the aspects required for an introduction. Such as, state of the art, summary of results, GAP, contributions, etc. | Updated the manuscript. Introduction section. Section 1, page 4  Added a new section. Section 1.1, Page 7 |
| It should have a subsequent section called literature review and hypothesis setting. | Many thanks for the comment. Agreed.  Added a new section. Section 1.1, Page 8. We have named it as ‘the concept of life expectancy and its determinants’. If required, we are happy to name it literature review. |
| The results section should be only the presentation of the results and not a discussion there. This happens for example in line 336. | Many thanks for the comments.  Corrected this.  Section 3, page 17. |
| The article does not have a discussion section as required. In the one included by the authors there is no discussion of the results obtained and contrast with those of authors of previous research. | Agree.  Updated the discussion section in the revised version. Added all the required references.  Section 4, page 24—29. |
| The conclusion section is very weak. It is necessary to indicate the main contributions of the research, as well as the possible limitations to the scope and future lines of research arising from this article. | Many thanks for the comments. Agree.  Contributions are noted on pp.7-8 and p. 28.  We have added one paragraph explaining limitations at the end of paper, pp 29-30. |
| In view of the above, I consider that the article requires major changes in order to be published. | Major revision is done. Thanks a lot. |

We sincerely hope that you will be kind enough to accept our revised version and publish the paper in your journal.

Best regards

Authors of the paper.

**Enclosures:**

1. Track changed manuscript
2. Clean manuscript
