# Supplementary material for: Determinants of life expectancy in most polluted countries: Exploring the effect of environmental degradation
Source: PLoS One. 2022 Jan 21;17(1):e0262802. doi: 10.1371/journal.pone.0262802 (PMC8782287; doi:10.1371/journal.pone.0262802)
Supplement: Attachment — Submitted filename: Response to Reviewer and Editor (R2)- Plos One.docx [file pone.0262802.s005.docx]

04 January 2022

María del Carmen Valls Martínez, Ph.D.
Academic Editor
PLOS ONE

Re: Second Revised version of the paper entitled *Determinants of Life Expectancy in Most Polluted Countries: Exploring the Effect of Environmental Degradation* **----***Response to the Editor and Reviewers.*

**PONE-D-21-32920 R1**

PLOS ONE

Dear Editor,

We are grateful to you for providing us the opportunity to revise the paper again (second revision) based on the recommendations of you and the reviewers. We are especially thankful to our respected reviewers for their thoughtful and constructive feedback on the paper. We very much appreciate for the time they spent on the paper. We have tried our best to accommodate/address all the suggestions they made in the paper. Because of their help, and our subsequent actions on their feedback, we believe the quality of the paper is now much improved. Their Comments and our Actions/Responses are noted below in detail for your kind consideration.

| **Editor’s Comments** | **Our Action and Responses** |
| --- | --- |
| Please review your reference list to ensure that it is complete and correct. If you have cited papers that have been retracted, please include the rationale for doing so in the manuscript text, or remove these references and replace them with relevant current references. Any changes to the reference list should be mentioned in the rebuttal letter that accompanies your revised manuscript. If you need to cite a retracted article, indicate the article’s retracted status in the References list and also include a citation and full reference for the retraction notice. | Thanks for the comment. Reference list is updated with new citations. These are reflected in the main manuscript and in the Reference List. |
| Additional Editor Comments (if provided):  The paper has been substantially improved after the revision. However, the final considerations made by reviewer 1 should be taken into account. | Thanks for the comment. In this final version, the reviewer 1’s new comments have been properly addressed. |
| **Reviewers’ Comments and Authors’ Actions /Responses** | |
| **Reviewer’s Comments** | **Our Action and Responses** |
| 1. If the authors have adequately addressed your comments raised in a previous round of review and you feel that this manuscript is now acceptable for publication, you may indicate that here to bypass the “Comments to the Author” section, enter your conflict of interest statement in the “Confidential to Editor” section, and submit your "Accept" recommendation.  Reviewer #1: All comments have been addressed  Reviewer #2: All comments have been addressed | Thanks Reviewers. |
| 2.Is the manuscript technically sound, and do the data support the conclusions?  The manuscript must describe a technically sound piece of scientific research with data that supports the conclusions. Experiments must have been conducted rigorously, with appropriate controls, replication, and sample sizes. The conclusions must be drawn appropriately based on the data presented.  Reviewer #1: Yes  Reviewer #2: Yes | Many thanks for the comment. |
| 3. Has the statistical analysis been performed appropriately and rigorously?  Reviewer #1: Yes  Reviewer #2: Yes | Many thanks for the comment. |
| 4. Have the authors made all data underlying the findings in their manuscript fully available?  The [PLOS Data policy](http://www.plosone.org/static/policies.action#sharing) requires authors to make all data underlying the findings described in their manuscript fully available without restriction, with rare exception (please refer to the Data Availability Statement in the manuscript PDF file). The data should be provided as part of the manuscript or its supporting information, or deposited to a public repository. For example, in addition to summary statistics, the data points behind means, medians and variance measures should be available. If there are restrictions on publicly sharing data—e.g. participant privacy or use of data from a third party—those must be specified.  Reviewer #1: No  Reviewer #2: Yes | Many thanks for the comment.  All the raw data used in this study are publicly and freely available. Please refer to the relevant websites noted in the manuscript. However, we have now attached our structured data set that is used for estimation purpose. |
| 5. Is the manuscript presented in an intelligible fashion and written in standard English?  PLOS ONE does not copyedit accepted manuscripts, so the language in submitted articles must be clear, correct, and unambiguous. Any typographical or grammatical errors should be corrected at revision, so please note any specific errors here.  Reviewer #1: Yes  Reviewer #2: Yes | Many thanks for the comment. |
| 6. Review Comments to the Author  Please use the space provided to explain your answers to the questions above. You may also include additional comments for the author, including concerns about dual publication, research ethics, or publication ethics. (Please upload your review as an attachment if it exceeds 20,000 characters)  Reviewer #1: Once again, I would like to thank the editorial board of Plos One for allowing me to review for such a renowned scientific publication. Focusing on my revision of the article (PONE-D-21-32920R1), below is my decision:  Accept | Many thanks for the comment. |
| **Reviewer 1:** I must recognize the effort made by the authors to rework this paper, which I consider has gained a lot of added value after implementing the changes and proposals for improvement suggested by the reviewers, therefore, congratulations to the authors for the work done. In any case, I would ask the authors to take into account the following points. 1. Use the "p" of the p-values always in italics. 2. Check if there is an error in Table 2 (Z-t-tilde-bar????). 3. It seems that the new references used do not appear in the list of final references. Please, check it. 4. The answer given for not using the KPSS test, believe me, is not valid at all. Keep this point in mind for future scientific works. Likewise, I suggest on a personal level that you do not support your views exclusively on Wikipedia. 5. I would consider that a greater emphasis on characterizing why in several non highly industrialized countries, which in many cases are geographically close, such high episodes of environmental pollution occur would have been mandatory.  Please bear in mind the points I have just made. In any case, congratulations.  With my best wishes in your personal and academic life, | Many thanks for the comments.  The further specific points are helpful, and our actions and responses are as follows:   1. "p" of the p-values is used in italic. 2. Corrections are made in Table 2. 3. Reference list is updated with new citations. These are reflected in the main manuscript and in the Reference List. 4. Thanks for your opinion. We will explore more about the   KPSS test for our future research keeping your advice in mind.   5. The fact is that the pollution level is high in these countries for whatever the reason. This is supported by data (from reliable sources) presented and explained in Appendix-B. Since our aim is to explore the link between life expectancy and pollution level, that is why these 31 most polluted countries were selected for the study though some of these are non-highly industrialized countries. A detailed description on “why pollution level is high in these countries” was not possible due to space consideration of the manuscript. However, a country-specific study can explain this in detail, which will be our future research agenda.   Thanks, reviewer, for your best wishes. |
|  |  |
| **Reviewer 2: Comments** | **Our Action and Responses** |
| Reviewer #2: I was pleased to see that the authors have taken my recommendations into account. This has allowed the article to improve significantly compared to the first evaluated version. For this reason, I consider the article suitable for publication. | Many thanks for the comment. |

We sincerely hope that you will be kind enough to accept our revised version and publish the paper in your journal.

Best regards

Authors of the paper.

**Enclosures:**

1. Track changed manuscript
2. Clean manuscript
3. Data file
